# Assessment of insert sizes and adapter content in fastq data from NexteraXT libraries

**DOI:** 10.3389/fgene.2014.00005

**Published:** 2014-01-30

**Authors:** Frances S. Turner

**Affiliations:** ARK-Genomics, Genetics and Genomics, Roslin Institute, University of EdinburghEaster Bush, UK

**Keywords:** Nextera, fastq, insert, adapter, Illumina

## Abstract

The Illumina NexteraXT transposon protocol is a cost effective way to generate paired end libraries. However, the resulting insert size is highly sensitive to the concentration of DNA used, and the variation of insert sizes is often large. One consequence of this is some fragments may have an insert shorter than the length of a single read, particularly where the library is designed to produce overlapping paired end reads in order to produce longer continuous sequences. Such small insert sizes mean fewer longer reads, and also result in the presence of adapter at the end of the read. Here is presented a protocol to use publicly available tools to identify read pairs with small insert sizes and so likely to contain adapter, to check the sequence of the adapter, and remove adapter sequence from the reads. This protocol does not require a reference genome or prior knowledge of the sequence to be trimmed. Whilst the presence of fragments with small insert sizes may be a particular problem for NexteraXT libraries, the principle can be applied to any Illumina dataset in which the presence of such small inserts is suspected.

## INTRODUCTION

**FIGURE 1:**
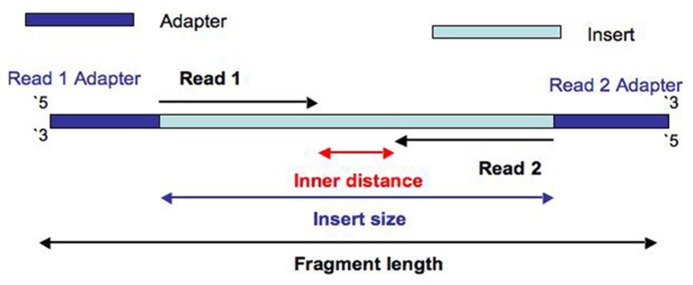
**Diagram to show the construction of a fragment with an insert size is longer than the length of both reads**.

**FIGURE 2:**
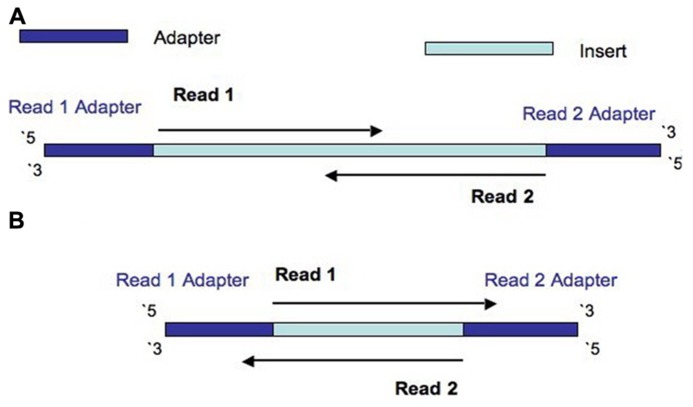
**Diagram to show the construction of a fragment with an insert size is less than the length of both reads, but more than the length of a single read (A), and the construction of a fragment with an insert size is less than the length of a single read (B)**.

The Illumina NexteraXT transposon protocol is a cost effective way to generate paired end libraries. Transpososomes are used to fragment DNA to be sequenced and add adapter sequences in a single step (known as tagmentation). The DNA between adapter the sequences is the insert. The length of this sequence is known as the insert size (not to be confused with the inner distance between reads, see **Figure 1**). The resulting insert size is highly sensitive to the concentration of DNA used. The variation of insert sizes is often large and the average size difficult to control. This can result in a proportion of fragments with an insert size of less than the length of a single read. Typically Illumina paired end reads have an insert longer than the combined length of both reads (see **Figure 1**). Using the latest Illumina platform, the MiSeq, paired end reads of 250 (and recently even 300) base pairs (bp) can be obtained. It can be useful to produce fragments with an insert size of less than the combined length of two reads, allowing the two ends to overlap (see **Figure 2A**). This allows the creation of even longer reads, which may be useful for some types of analysis, particularly *de novo* assembly, metagenomic studies or 16s ribosomal RNA analysis. It also helps to deal with the lower base quality that is typically found at the end of long reads, as the ends of the read are sequenced twice. However, when aiming for an insert size of less than the length of the two reads, the broad range of insert sizes found in NexteraXT libraries means that there may be some fragments with an insert size less than the length of a single read. Such fragments are less useful than those with longer inserts as they do not extend reads beyond their original length. Short inserts also have consequences for the presence of adapter in the reads. Reads will contain the entire insert and run into the adapter on the opposite end of the fragment (see **Figure 2B**). Therefore the 3′ end of read 1 will contain the reverse complement of the adapter attached to read 2, and vice versa.

A measurement of the numbers of read pairs with an insert size of less than the length of a single read is a useful step in the quality control of fastq data from NexteraXT libraries (or any other library in which the presence of small insert sizes is suspected). The detection of such read pairs may indicate the need to trim adapter sequences. As these read pairs are less useful than those with longer inserts, the number of read pairs with an insert size greater than the length of a single read may provide a better indication of the amount of useable data, rather than the total number of read pairs. Unexpectedly high levels of read pairs with short inserts may indicate problems with laboratory protocols, so this quality control step can also be useful to provide feedback to the laboratory. The proportion of read pairs with short inserts that can be found in a dataset before the laboratory may need to be alerted, or the data may be considered inadequate for the planned analysis, will depend on the individual circumstances of the experiment. The proportion of fragments with small inserts that may be expected in a library will vary according to the exact details of the library preparation. The extent to which a certain proportion of read pairs with short inserts will impact on the data analysis will depend upon the total number of read pairs generated, and the aims of the analysis. Guidelines regarding the level of short inserts in a dataset that may be considered problematic are beyond the scope of this paper.

**FIGURE 3:**
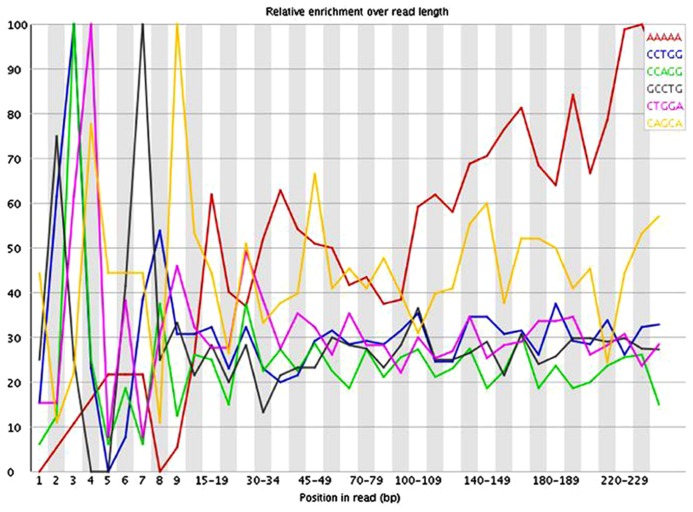
**FastQC plot of the relative enrichment of k-mers of five nucleotides along the length of the read for a single library in the dataset**.

**FIGURE 4:**
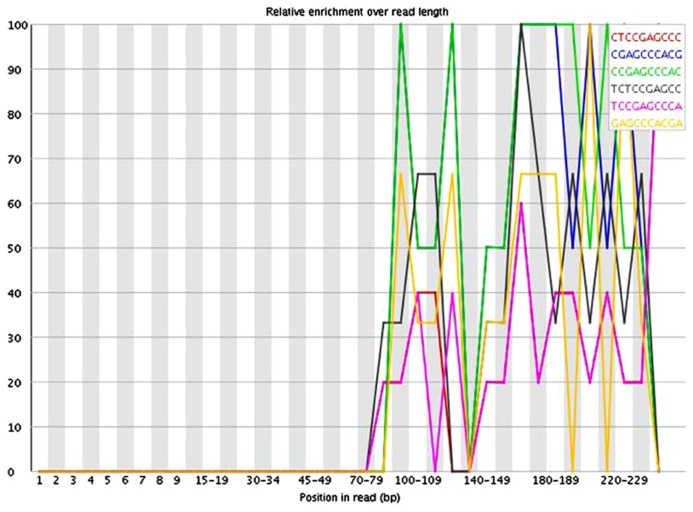
**FastQC plot of the relative enrichment of k-mers of 10 nucleotides along the length of the read for a single library in the dataset**.

The presence of these short inserts in a library may be reflected in a number of possible quality control metrics. Programs such as USEARCH (Edgar, 2010) can be used to scan for specified adapter sequences. K-mer plots produced by FastQC (Andrews, 2010) show the relative enrichment of k-mers along the length of the read. These can also provide indication that adapter sequences are present due to short inserts. The example in **Figure 3** shows a plot of the relative enrichment of k-mers of five nucleotides in a single library from the example dataset. It can be seen that a run of As occur toward the ends of the reads. This indicates the presence of fragments that have very small inserts, as a stretch of As typically occurs when a read has gone all the way through the adapter sequence and past the end of the fragment. **Figure 4** shows a plot of the relative enrichment of k-mers of 10 nucleotides from the same library. Using the longer k-mer length it can be clearly seen that k-mers belonging to the Nextera adapter sequences are enriched toward the end of the reads. Despite the existence of these other approaches, the measurement of insert sizes is a direct and easy to way to detect and quantify the level of short inserts in a dataset.

Here I show how insert sizes can be reliably measured independently of a reference genome sequence, and the reads likely to contain adapters identified. I proceed to show how the sequence of the adapters at the end of the read can be verified, and the adapter sequence trimmed from the read. The dataset used in this analysis was generated by sequencing 96 NexteraXT libraries prepared from *Escherichia coli* genomic DNA on the MiSeq platform to generate 250 bp end reads. A random sample of 10,000 reads was taken from each library.

## RESULTS

### MEASUREMENT OF INSERT SIZE

**FIGURE 5:**
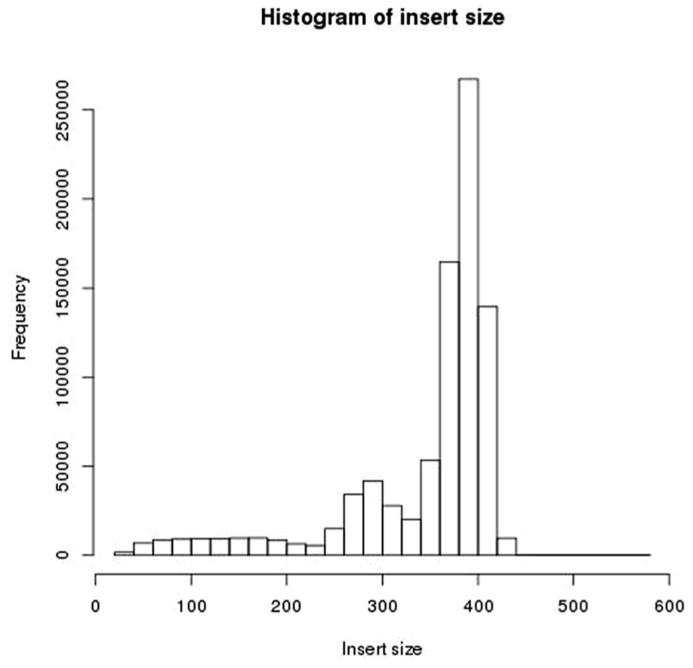
**Histogram of insert sizes of read pairs for a single library in this dataset**.

The insert size of a read pair can be reliably measured by mapping to a reference genome. The insert size distribution of a fastq dataset can be extracted directly from a sequence alignment format (SAM) file, providing a convenient way to identify the presence of fragments with excessively small insert sizes in a dataset. **Figure 5** shows a histogram of insert sizes measured by mapping read pairs from a typical library from this dataset to the reference genome. However this approach to measurement of insert size relies on both a reference genome sequence and accurate mapping of reads to this reference. Where the insert size is less than the combined length of both reads (see **Figure 2A**), it can be measured independently of a reference genome by attempting to overlap the two reads. FLASH (Magoč and Salzberg, 2011) can be used to overlap the 3′ ends of such reads. This insert size can then be derived, as the length of the contig produced will be equal to the insert size. Where the insert size is less than the length of a single read (see **Figure 2B**), the 5′ ends of the reads will overlap. FLASH attempts to overlap the 3′ end of the read, so will not overlap such reads. However FLASH will overlap the reverse (not the reverse complement) of the sequences, and so insert sizes less than the read length can be measured using this equation.

$$i=\left(r_{1}+r_{2}\right)-c$$

Where *i* is the insert size, *r*_1_ is length of read 1, *r*_2_ is the length of read 2, and *c* is the length of the contig produced by FLASH.

**FIGURE 6:**
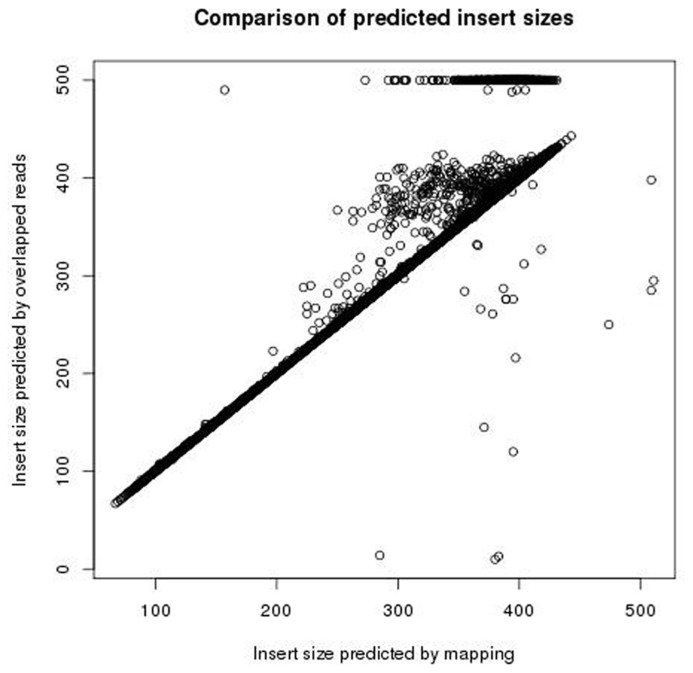
**Plot showing the insert sizes predicted using FLASH and the insert sizes predicted by mapping to the reference**.

The insert size was measured by both mapping read pairs the reference genome, and by using FLASH to overlap the reads (trying both the forward and reversed sequences). The two approaches give similar measurements of insert size. **Figure 6** shows a comparison of measures of insert sizes obtained by overlapping the reads, and measures of insert sizes obtained by mapping to the reference. For 92% of reads the difference between the measurements of insert size by the two methods is five bases or less. Where discrepancies between the two methods do occur, they seem to be the result of FLASH either failing to overlap reads pairs with an insert size of more than the length of a single read but less than the length of both reads, or under estimating the extent to which these reads overlap, and so over estimating the insert size. The two methods show few differences where the insert size is less than the length of a single read. Nine percent of read pairs were found to have an insert size of less than 250 bp (and so likely to contain adapter) after mapping to the reference genome, of which 98% were also found to have an insert size of less than 250 bp by overlapping the reversed sequence. Therefore the overlapping of reversed reads provides a reliable method for detecting small insert sizes, without the need for a reference genome.

FLASH is widely used, but other programs to overlap read pairs are available. Published methods include PEAR (Zhang et al., 2013), COPE (Liu et al., 2012), and PANDASeq (Masella et al., 2012). These programs may be substituted for FLASH in the protocol described here, but a comparison of the performance of these different methods is beyond the scope of this paper.

## IDENTIFICATION OF ADAPTER SEQUENCE

**FIGURE 7:**
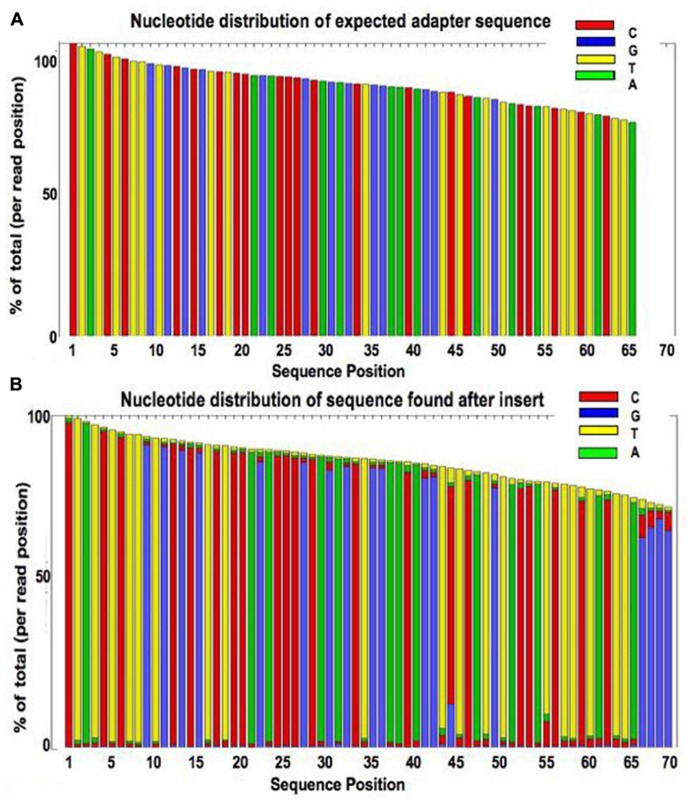
**Plot showing the base composition of the sequence calculated to occur after the insert (B) compared to a plot of the expected sequence of the Nextera adapter (A)**.

Just as using FLASH to overlap the reversed sequence of the reads can identify those likely to contain adapter, the same principle can be applied to identify the adapter sequence to be trimmed. The bases occurring after the *i* th position of a read (where *i* is the insert size as calculated in Section “Measurement of Insert Size”) come from the oligonuleotide attached to the insert. After overlapping the reversed sequences of read pairs, and recording the lengths of the contigs formed by each pair, the section of each read calculated to be after the insert was extracted in fastq format. Examination of the extracted sequence confirmed that this matched the expected sequence. For read 1 this is the reverse complement of the Nextera transposase sequences attached to read 2, followed by the reverse complement of the index, then the PCR primers. **Figure 7B** shows a plot of nucleotide distribution at each base for sequences extracted from of read 1 for a single library. The first 67 bases show virtually no variability, as it is all adapter sequence. Where the insert size was very short and the read went all the way through the adapter, a run of As is seen followed by apparently random sequence. **Figure 7A** shows the plot that would be expected if the extracted sequence was purely the expected adapter.

### COMPARISON OF INSERT SIZES TO LENGTH OF SEQUENCE CUT BY TRIMMING TOOLS

When the insert of a read can be accurately estimated, the number of bases of adapter to be trimmed can also be derived. Where the insert is less than the length of a single read, the adapter trimmed read should be equal to the length of the insert size. It is therefore possible to compare the performance of different adapter trimming algorithms based on estimated insert sizes. A number of tools are available to trim adapter sequences from reads. Some published tools include AlienTrimmer (Criscuolo and Brisse, 2013), cutadapt (Marcel, 2011), AdapterRemoval (Lindgreen, 2012), Btrim (Kong, 2011). A comparison of the performance of all available adapter trimming tools is beyond the scope of this paper. However, Cutadapt and AlienTrimmer performed well in a recent comparison of adapter trimming tools (Criscuolo and Brisse, 2013), and AlienTrimmer is designed to efficiently trim a number of possible sequences, so may be a good choice where there is some uncertainty regarding the adapter sequence to be trimmed. For these reasons AlienTrimmer and cutadapt were chosen for assessment of their performance on this dataset. AlienTrimmer searches sequences for all possible k-mers of a given adapter sequence, so its results can vary depending upon the size of k-mer used. The performance of AlienTrimmer with three different k-mer values was compared. The sensitivity and specificity of the two tools were measured. A true positive result was defined as a read for which mapping to the genome indicated an insert of <250 bases, and for which the adapter was trimmed to leave no more than five bases of putative adapter sequence. A false positive result was defined as a read trimmed at least five bases more than necessary, based on the predicted insert size. A true negative result was defined as a read with a predicted insert size of >250, which was not trimmed more than five bases. A false negative result was defined as a read with a predicted insert size of <250, that after trimming had more than five bases of putative adapter sequence. Sensitivity is defined as *tp*/(*tp* + *fn*), where *tp* is the number of true positives and *fn* is number of false negatives. Specificity is defined as *tn*/(*tn* + *fp*) where *tn* is the number of true negatives and *fp* is number of false positives. Results are shown in **Table 1**.

**Table 1:** Comparison of the specificity and sensitivity of two adapter trimming tools.

	Sensitivity	Specificity
Cutadapt	0.99	>0.999
Alien Trimmer *k* = 8	0.18	>0.999
Alien Trimmer *k* = 10	0.23	>0.999
Alien Trimmer *k* = 15	0.18	0.995

**FIGURE 8:**
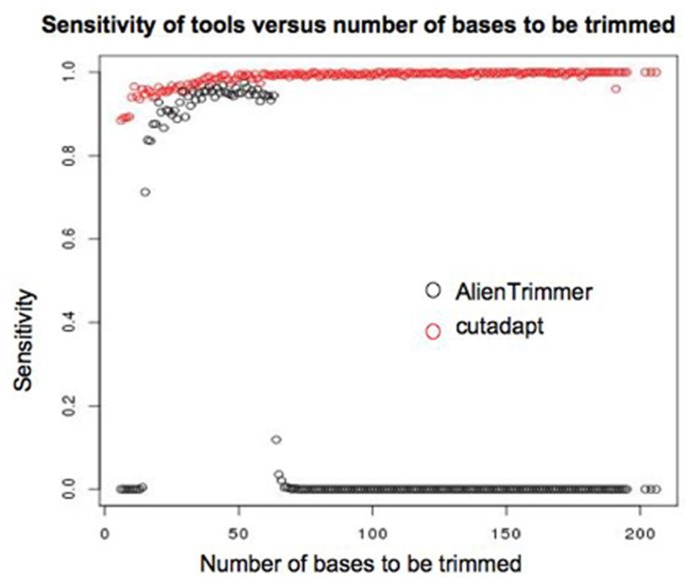
**Plot showing how the sensitivity of AlienTrimmer (with a k-mer value of 10) and cutadapt varies according to the length of adapter sequence expected to be trimmed based on insert size estimated by mapping**.

The low sensitivity of AlienTrimmer for this particular dataset seems to be at least partly due to its poor performance for reads that extended past the length of the fragment. **Figure 8** shows how the specificity of cutadapt and AlienTrimmer (for k-mer = 10) change depending of the number of bases that need to be trimmed. Where less than around 70 bases need to be trimmed, the performance of AlienTrimmer was more comparable to the cutadapt. Where more than 70 bases need to be trimmed AlienTrimmer was not successful. In these cases the read continues beyond the length of the fragment.

## MATERIALS AND METHODS

The dataset used here consists of 250 bp paired reads from *E. coli* sequenced on the MiSeq platform at ARK genomics. Demultiplexed fastq files were generated using CASAVA version 1.8. About 96 separate libraries were sequenced in one run. All analysis in this paper is performed using a dataset of 10,000 randomly chosen reads from each library. Libraries were prepared using standard dual index NexteraXT transposon protocol.

Read pairs were filtered by base quality. If after quality trimming using sickle (Najoshi, 2011) with a quality cutoff of 20, the combined length of both ends of the read would be less than 250 base pairs, the read pair was discarded. Read pairs where mapped to the K12_MG1655 genome using BWA (Li and Durbin, 2009) mem version 0.7.5 with default parameters.

Reads were overlapped to form contigs using FLASH version 1.2.2 with the parameters -r 250 f 370 s 80. Default parameters are not suitable for reads of this length and range of insert size.

To test the two trimming tools, reads passing the quality filter were trimmed using cutadapt version 0.9.4 and AlienTrimmer version 0.3.2. AlienTrimmer was used with three different k-mer values (8, 10, and 15). The sequence provided to the two tools to be trimmed off read 1 was CTGTCTCTTATACACATCTCCGAGCCCACGAGAC, which is the reverse complement of the Nextera transposase sequence attached to read 2 and the sequence to be trimmed of read 2 was CTGTCTCTTATACACATCTGACGCTGCCGACGA which is the reverse complement of the Nextera transposase sequence attached to read 1 (Oligonucleotide sequence 2007–2012 Illumina, Inc. All rights reserved). The comparison of number of bases trimmed to insert size measured by mapping to the reference was based only on read pairs with high quality mappings. Reads with mapped with a mapping quality of less than 30, or that had split hits, were not included in the comparison.

## DISCUSSION

Paired end reads with an insert size of less than the length of a single read contain less information than read pairs with longer insert sizes. If they occur at significant levels, the amount of useable sequence in a dataset will be reduced. Such reads will also contain adapter sequences, which may need to be trimmed they as can negatively impact on some types of analysis. The detection of these reads is therefore a necessary part of quality control for any library likely to have small insert sizes. Such short inserts can be measured using FLASH (or a similar program) to overlap the reversed sequence of the paired reads. This allows for a very fast assessment of the number of read pairs in a dataset that have short inserts, without the need for a reference sequence. Read pairs, that FLASH is able to overlap in the reversed sequence should have adapter sequence at the ends. As the length of the insert can be calculated from the length of the individual reads and overlapped read, it is possible to calculate how far each read continued past the insert. It is also possible to confirm the sequence of the oligonucleotide the read covered after the insert.

The sequence after the insert may need to be removed as it can affect analysis, in particular *de novo* assembly. If there is any uncertainty about the adapter sequence that needs to be trimmed, the sequence of a range of possible Illumina oligonucleotides could be provided to an adapter trimmer tool. AlienTrimmer is designed to be able run quickly, even given a long list of possible sequences to trim (Criscuolo and Brisse, 2013). However, the comparison of these tools in Section “Comparison of Insert Sizes to Length of Sequence Cut by Trimming Tools” suggests that AlienTrimmer failed to trim adapter from reads that continued past the adapter sequence. Therefore AlienTrimmer would not be a good choice for datasets with many such reads. Cutadapt did well even where the read continued past the adapter sequence. Cutadapt can also be provided with a long list of possible contaminant sequences (although this would cause it to run more slowly). Both tools (along with the wide range of other tools to detect and remove adapter sequence) need to be provided with a sequence to be trimmed (or a list of possible sequences to be trimmed). They would therefore fail if there is unexpected sequence occurring after the insert. Whilst the adapter sequence to be removed would generally be known, there may be some situations in which it is useful confirm this. In the context of data quality control a sequencing laboratory it would be useful to know if the sequence occurring after the insert is not the expected sequence, as this could indicate a problem with library construction. When working with data obtained from a public repository it may be difficult to obtain information regarding the adapters used.

This method to predict the position and sequence of the adapter to be trimmed cannot be applied to Nextera long mate pair (LMP) libraries. In these libraries the adapter sequence to be trimmed may occur anywhere in the read rather than at the 3′ end. Therefore the position of adapter in a read cannot be predicted from the length of contig formed when the 5′ ends of a read pair overlap. NextClip (Leggett et al., 2013) is a tool that can be used to detect and remove adapter sequences in LMP libraries.

## CONCLUSION

The protocol described in this paper to detect and remove adapter sequences could in principle be applied to any small insert size Illuminia paired end library, uses readily available tools, and does not require prior knowledge of the adapter sequence or access to a reference genome.

## Conflict of Interest Statement

The author declares that the research was conducted in the absence of any commercial or financial relationships that could be construed as a potential conflict of interest.
